# Neurosurgical management of geriatric patients with traumatic brain injury in a medium-developed Chinese city: a recent-years overview

**DOI:** 10.3389/fneur.2025.1691924

**Published:** 2025-11-07

**Authors:** Xiao-ting Fan, Hai-ying Zhao, Chuan-fu Wu, Bao-xiang Gao, Jian Li, Yan Xu, Yong-gang Lian, Sheng-ji Wang

**Affiliations:** 1 Department of Neurosurgery ICU, Linyi People's Hospital Affiliated to Shandong Second Medical University, Linyi, Shandong, China; 2 Emergency Department, Linyi People's Hospital Affiliated to Shandong Second Medical University, Linyi, Shandong, China; 3 Center of Health Data Science, Linyi People's Hospital Affiliated to Shandong Second Medical University, Linyi, Shandong, China; 4 Shandong Open Laboratory of Data Innovation Application, Linyi, Shandong, China

**Keywords:** traumatic brain injury, head injury, neurosurgery, clinical outcome, geriatric patients, elderly

## Abstract

**Background:**

Traumatic brain injury (TBI) is a leading cause of morbidity and mortality worldwide, with outcomes influenced by age and comorbidities. This study aimed to compare the clinical characteristics, surgical management, and outcomes of elderly and younger TBI patients.

**Methods:**

Between 2017 and 2022, 1,260 TBI patients admitted to our hospital were included and categorized into younger (18–59 years) and elderly (≥60 years) groups. Demographic data, injury mechanisms, types of brain trauma, surgical interventions, and discharge outcomes were analyzed.

**Results:**

Elderly patients had higher rates of comorbidities, with traffic accidents as the leading cause of injury and falls predominating in those aged ≥75 years. They showed a higher proportion of subdural hemorrhages, higher preoperative GCS scores, and required more mechanical ventilation and tracheostomy but underwent fewer decompressive craniectomies. In-hospital mortality was slightly lower in the elderly group, whereas rates of vegetative state and moderate-to-severe disability were higher, reflecting age-related differences in clinical outcomes and surgical management.

**Conclusions:**

Age significantly influences the clinical presentation, management strategies, and functional outcomes of TBI patients. Tailored surgical and postoperative care are crucial for optimizing survival and quality of life in elderly patients.

## Introduction

Traumatic brain injury (TBI) is induced by external mechanical forces, including direct impact, rapid acceleration–deceleration, or penetrating head injury, and represents a major global health concern (1). According to a global epidemiological study conducted in 2016, the annual incidence and prevalence of traumatic brain injury (TBI) in the United States were 333 and 605 per 100,000 population, respectively. In China, the corresponding figures were 313 and 742 per 100,000 population, highlighting the substantial burden of TBI in both countries (2). Moreover, due to its large population, the absolute number of TBI cases in China surpasses that of most other countries, resulting in a considerable social and familial burden (3).

Globally, population aging further amplifies this challenge. In 2019, the number of people aged 60 years or older reached one billion, and this figure is projected to rise to 1.4 billion by 2030 and 2.1 billion by 2050 (4). This unprecedented demographic transition, particularly pronounced in developing countries, is expected to be accompanied by a marked increase in injuries and injury-related mortality among the elderly.

At present, research on neurosurgical approaches and prognostic outcomes in elderly patients with TBI remains limited, particularly in developing regions (5). There is an urgent need for further investigations in this underexplored yet rapidly expanding demographic to guide clinical practice and ensure adequate healthcare resource allocation.

Linyi People's Hospital, a Class A tertiary care center in Shandong Province, functions as the primary neurosurgical referral institution for Linyi, the largest developing city in the province, with a population of more than 11 million (6). Against this backdrop, we designed a retrospective study to evaluate the clinical characteristics, surgical interventions, and in-hospital outcomes of elderly patients undergoing neurosurgical treatment for TBI at our institution in recent years.

## Methods

### Study material

The present study was a retrospective analysis of patients with acute traumatic brain injury (TBI) who underwent neurosurgical treatment at Linyi People's Hospital between 2017 and 2022. Eligible patients were those consecutively admitted within 24 h of injury, aged 18 years or older, with cerebral computed tomography (CT) demonstrating intracranial signs of trauma. Clinical data were extracted from the hospital's electronic medical records and included demographic variables (age, sex), injury-related factors (cause of injury, associated injuries), medical history (comorbidities, prior conditions), preoperative neurological status [Glasgow Coma Scale (GCS) score], treatment details, and outcomes assessed by the Glasgow Outcome Scale (GOS) at discharge.

Inclusion criteria: Patients who underwent neurosurgical treatment during hospitalization, including decompressive craniectomy (DC), craniotomy, skull burr holes/cranial trephination and drainage, intracranial pressure (ICP) catheter implantation, and skull fracture repair.

Exclusion criteria: (1) Patients who did not undergo cranial surgery during hospitalization; (2) Patients who died within 24 h of admission; (3) Patients younger than 18 years; (4) Patients with incomplete electronic clinical information.

In this study, ICP monitoring catheters included both intraventricular and intraparenchymal devices. Cranial trephination and drainage included external ventricular drainage (EVD) and evacuation/drainage of intracranial hematomas. Drainage catheters can be placed epidurally, subdurally, within a hematoma, or intraventricularly. Skull fracture repair procedures included reduction of skull fracture, cranioplasty, elevation and debridement, dural repair, and fixation.

### Statistical analysis

The distribution of variables was assessed using the Kolmogorov–Smirnov test. Continuous variables that did not follow a normal distribution were analyzed using the Mann–Whitney *U* test and presented as medians with interquartile ranges (IQR). Categorical variables were expressed as absolute numbers and percentages, and comparisons were made using the χ^2^ test when no inherent order was present. A *p*-value of < 0.05 was considered statistically significant. All statistical analyses were performed with IBM SPSS Statistics for Windows, version 23.0 (IBM Corp., Armonk, NY, USA).

## Results

**Figure 1:**
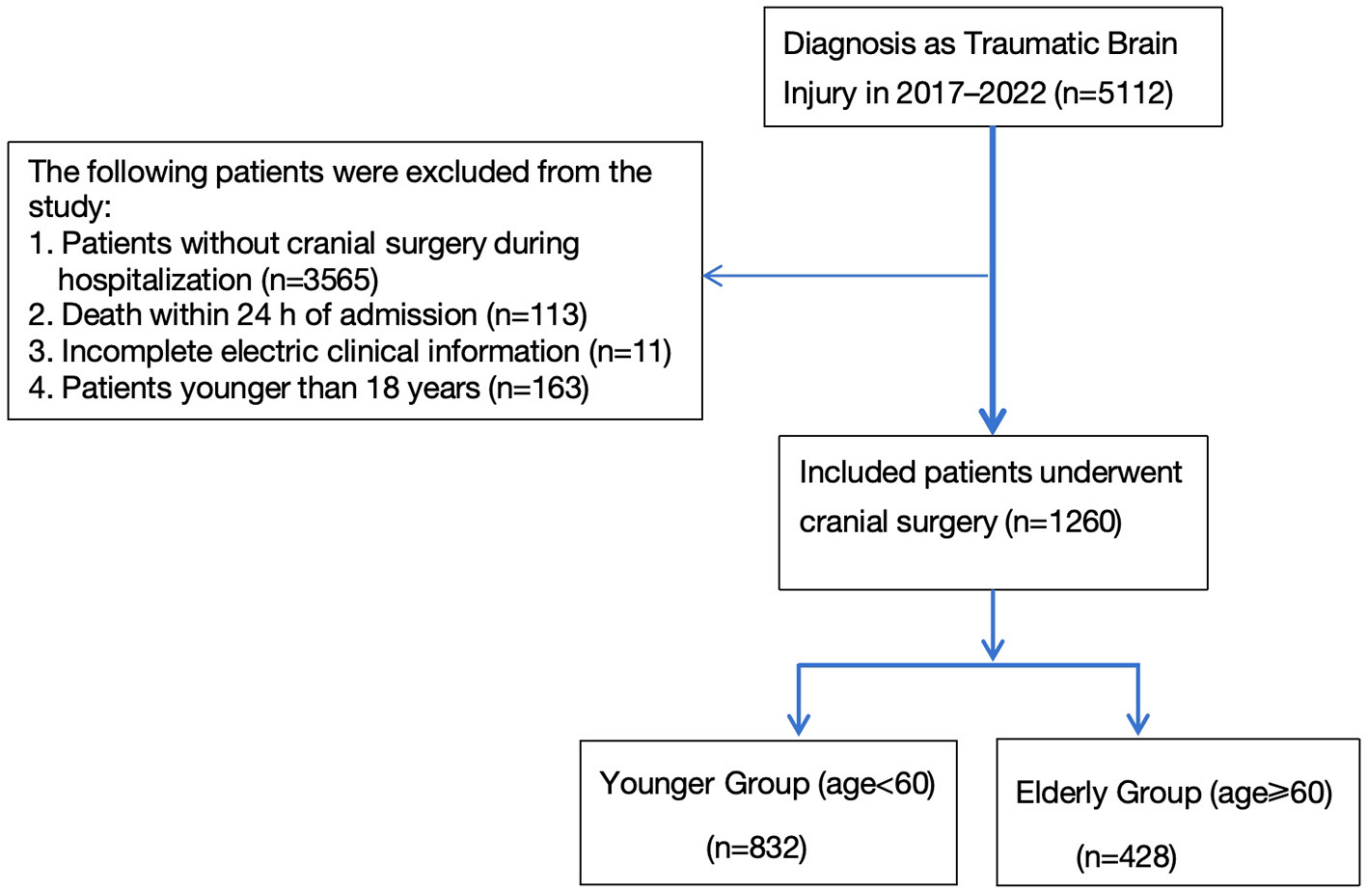
Patient inclusion and exclusion flowchart.

As shown in Figure 1, from 2017 to 2022, a total of 5,112 TBI patients were admitted to our hospital. After applying exclusion criteria, 1,260 patients were included in the analysis. Patients were divided into two age groups: the Younger Group (18 ≤ age < 60) and the Elderly Group (age ≥ 60). Glasgow Outcome Scale (GOS) scores at discharge were recorded for all patients. Demographic characteristics and clinical management for the two groups are summarized in Table 1. Compared with younger patients, the elderly group had a lower proportion of male patients but a significantly higher prevalence of diabetes, hypertension, and prior stroke.

**Table 1:** Demographic characteristics and clinical treatment of the two groups.

**Variables**	**Younger group (*n* = 832)**	**Elderly group (*n* = 428)**	***p-*Value**
Gender (male), *n* (%)	643 (77.3)	304 (71.0)	0.015
Diabetes, *n* (%)	37 (4.4)	42 (9.8)	< 0.001
Hypertension, *n* (%)	71 (8.5)	95 (22.2)	< 0.001
Cerebral stroke, *n* (%)	13 (1.6)	16 (3.7)	0.015
Aetiology, *n* (%)			< 0.001
Road traffic injuries	367 (44.1)	237 (55.4)	
Fall	410 (49.3)	178 (41.6)	
Violence	55 (6.6)	13 (3.0)	
**TBI subtype**, ***n*** **(%)**			
CC	703 (84.5)	340 (79.4)	0.024
SDH	664 (79.8)	383 (89.5)	< 0.001
SAH	656 (78.8)	324 (75.7)	0.203
EDH	415 (49.9)	82 (19.2)	< 0.001
SF	352 (42.3)	110 (25.7)	< 0.001
Traumatic epilepsy, *n* (%)	38 (4.6)	23 (5.4)	0.528
Preoperation GCS	6 (5.9)	8 (6.10)	0.012
Cerebral herniation, *n* (%)	157 (18.9)	73 (17.1)	0.43
Systemic hypotension, *n* (%)	45 (5.4)	31 (7.2)	0.063
Hypoxia, *n* (%)	106 (12.7)	68 (15.9)	0.034
Cardiopulmonary resuscitation, *n* (%)	14 (1.7)	14 (3.3)	0.07
**Concomitant injuries**, ***n*** **(%)**			
Chest	257 (30.9)	141 (32.9)	0.457
Abdomen	14 (1.7)	7 (1.6)	0.951
Limbs	50 (6.0)	18 (4.2)	0.18
Spine	115 (13.8)	58 (13.6)	0.895
**Neurosurgical treatment**, ***n*** **(%)**			
DC	430 (51.7)	190 (44.4)	0.014
ICP monitoring	498 (59.9)	265 (61.9)	0.479
Cranial trephination and drainage	314 (37.7)	224 (52.3)	< 0.001
Craniotomy	295 (35.5)	128 (29.9)	0.059
Skull fracture repair surgery	217 (26.1)	67 (15.7)	< 0.001
Unplanned second surgery	52 (6.2)	26 (6.1)	0.521
Ventilator, *n* (%)	27 (3.2)	26 (6.1)	0.035
Tracheotomy, *n* (%)	30 (3.6)	30 (7.0)	0.007
Hospital stay, days	29 (25,30)	28 (24.29)	0.874
In-hospital mortality, *n* (%)	141 (16.9)	60 (14.1)	0.039
**Hospital cost**, ¥			
Medical care fees	62,880.840 (38,078.943, 84,775.105)	59,431.845 (38,574.280, 66,840.705)	0.71
Medicine expenses	47,278.685 (27,691.293, 59,220.273)	41,976.510 (28,653.140, 57,743.735)	0.339
Surgery fee	28,546.155 (21,586.573, 37,982.695)	26,850.605 (16,165.480, 35,694.715)	< 0.001
Cost of anti-infective drugs	6,024.095 (2,556.635, 12,578.475)	6,556.570 (2,152.333, 102,77.755)	0.744

GCS, Glasgow Coma Scale; CC, cerebral contusion; SAH, traumatic subarachnoid hemorrhage; SDH, acute subdural hematoma; SF, skull fractures (including the base of skull fractures); EDH, acute epidural hematoma; DC, decompressive craniectomy; ICP, intracranial pressure.

Traffic accidents were the leading cause of injury in the elderly group (55.4%), followed by falls (41.6%). To further examine injury mechanisms, the elderly group was subdivided into four age subgroups, as shown in Table 2. Notably, falls were the primary cause of injury only in patients aged 75 years or older.

**Table 2:** Comparison of etiology of TBI in different age groups.

**Variables**	**60–64 years old (*n* = 143)**	**65–69 years old (*n* = 140)**	**70–74 years old (*n* = 76)**	**≥75 years old (*n* = 69)**	***p-*Value**
**Aetiology**, ***n*** **(%)**			
Road traffic injuries	73 (51)	83 (59.3)	48 (63.2)	33 (47.8)	0.292
Fall	63 (44.1)	54 (38.6)	27 (35.5)	34 (49.3)	
Violence	7 (4.9)	3 (2.1)	1 (1.3)	2 (2.9)	

**Figure 2:**
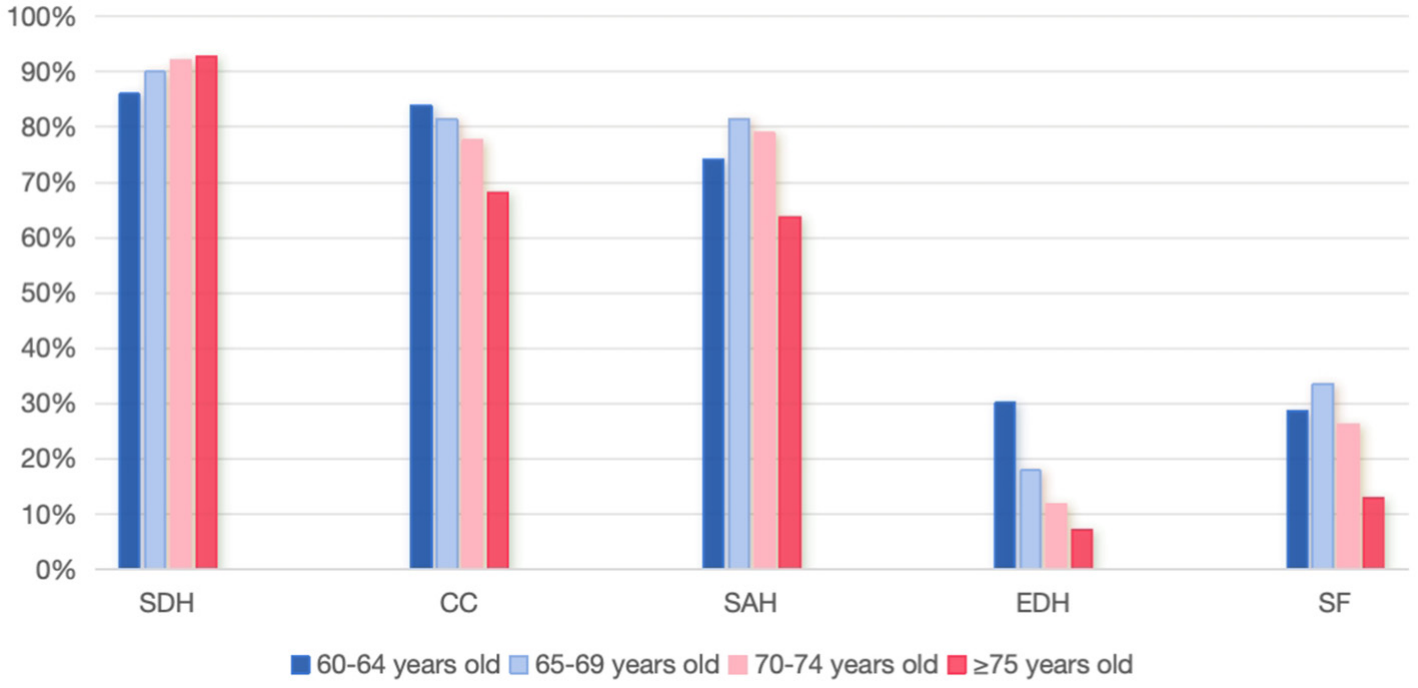
Comparison of brain trauma types in different age groups.

Regarding the types of brain trauma, the elderly group exhibited lower rates of brain contusions, epidural hematomas, and skull/skull base fractures, but a higher proportion of subdural hemorrhages compared with the younger group (Table 1). The comparison of traumatic brain injury types among different age groups is presented in Figure 2. Preoperatively, elderly patients had higher median GCS scores (median 8). No significant differences were observed between the groups in rates of brain herniation or post-traumatic epilepsy. However, the elderly group had higher rates of hypoxemia (15.9 vs. 12.7%) and mechanical ventilation (6.1 vs. 3.2%). Rates of cardiopulmonary resuscitation and hypotension were also higher in the elderly group, though these differences were not statistically significant.

There were no significant differences in concomitant injuries between the two groups. Regarding surgical management, the elderly group had lower rates of decompressive craniectomy (DC) and skull fracture repair procedures (44.4 vs. 51.7%; 15.7 vs. 26.1%) but a higher rate of cranial trephination and drainage (52.3 vs. 37.7%), with statistically significant differences. No significant differences were observed in ICP monitoring, craniotomy, or unplanned secondary surgeries.

In-hospital mortality was lower in the elderly group than in the younger group (14.1 vs. 16.9%), whereas the rate of tracheostomy was higher (7 vs. 3.6%). Length of hospital stay was similar between the groups. Surgical costs were lower in the elderly group, while other hospitalization costs (including medical care, medications, and anti-infective drugs) were comparable.

**Figure 3:**
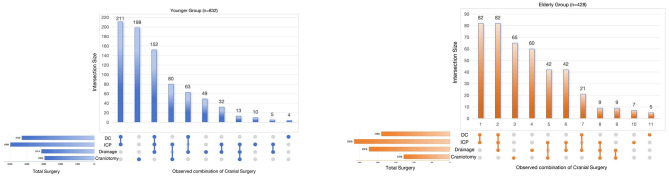
Selection and combination of cranial surgery types in the two groups. UpSet plots were used to display cranial surgery types. Four primary cranial surgery types about TBI lesion (DC decompressive craniectomy; ICP, intracranial pressure monitoring; Drainage, cranial trephination and drainage; craniotomy).

Surgical approaches were tailored to patient conditions, such as DC combined with ICP catheter implantation or DC combined with cranial trephination and drainage. The comparative surgical characteristics between the two groups are shown in Figure 3.

**Figure 4:**
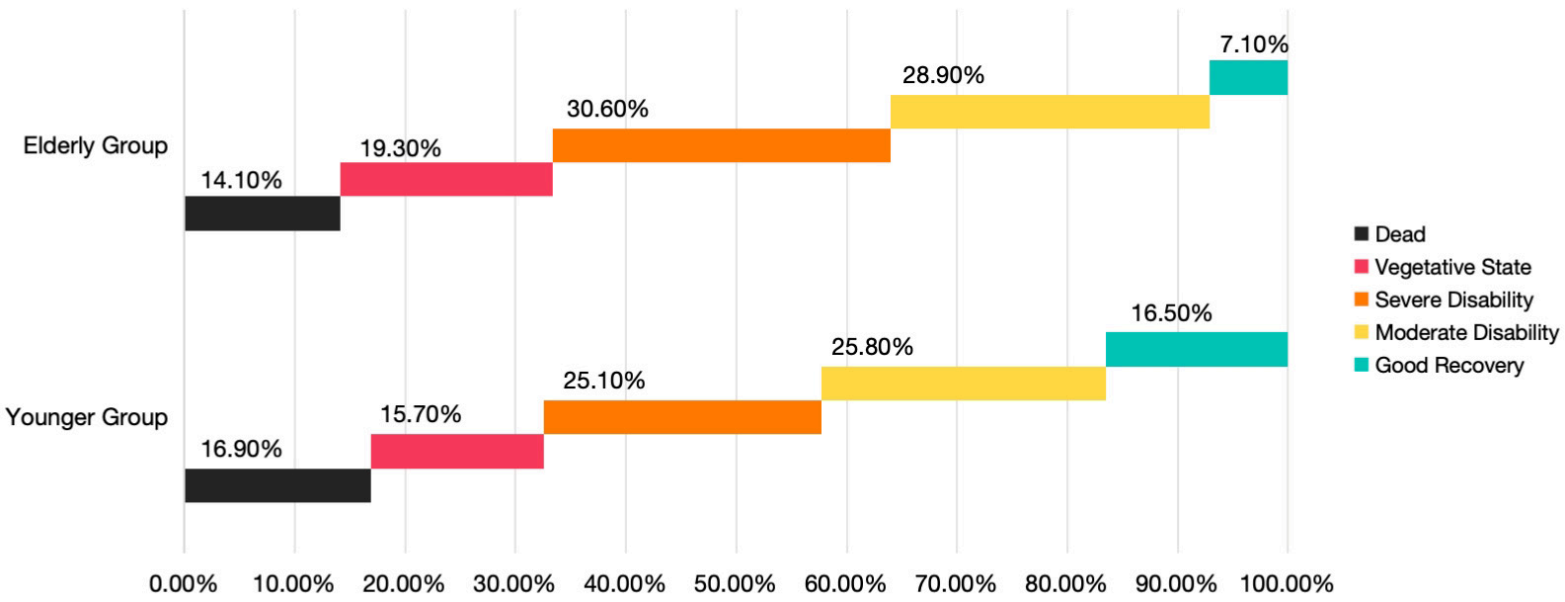
Comparison of percentage of patients between the two groups based on GOS class at hospital discharge.

Prognostic comparisons based on discharge GOS scores are shown in Figure 4. The elderly group exhibited lower mortality and complete recovery rates than the younger group, whereas the proportion of patients in a vegetative state or with moderate-to-severe disability was higher in the elderly group.

## Discussion

Our study analyzed the demographic characteristics, surgical approaches, and prognostic outcomes of elderly patients with acute TBI who underwent neurosurgical intervention. We found that elderly patients were more likely to receive minimally invasive procedures of shorter duration. Although their short-term mortality rate was lower, these patients exhibited a higher likelihood of moderate to severe postoperative disability, necessitating intensive care and prolonged rehabilitation. These findings provide real-world, evidence-based insights to inform future management strategies for elderly patients with TBI.

In our cohort, the main reason for injury among elderly patients was road traffic accidents. By contrast, a study conducted in the United Kingdom by Kehoe et al. (7) reported that more than 80% of elderly patients (≥65 years) sustained TBIs as a result of falls. Similarly, a large epidemiological survey in China indicated that incidental falls (*n* = 1,044, 43.23%) were the leading cause of TBI in old patients (8). These observations highlight a distinct difference in the predominant mechanisms of injury between developed and developing regions, underscoring the need for region-specific prevention and intervention strategies.

In this study, the incidence of subduralhemorrhage among elderly TBI patients increased with advancing age. This high frequency is thought to be related to age-associated brain atrophy, which enlarges the subdural space and predisposes elderly individuals to such lesions (9).

Elderly individuals frequently present with preexisting conditions such as hypertension and diabetes, making them more vulnerable to post-TBI complications including asphyxia, hypoxia, and shock. It is well-recognized that aging reduces physiological reserve, and the presence of comorbidities—as well as their ongoing treatments—can significantly influence the disease course and clinical outcomes (10). Moreover, the management of blood pressure in elderly hypertensive patients following TBI remains a subject of debate (11, 12). In particular, the optimal systolic blood pressure (SBP) threshold has recently been questioned, especially in developing countries where advanced monitoring tools such as intracranial perfusion pressure and cerebral blood flow measurements are not routinely available (13).

In our study, elderly patients underwent fewer decompressive craniectomies (DC) and craniotomies compared with younger patients. For this population, surgeons appeared to favor shorter, less invasive procedures aimed at rapidly alleviating life-threatening conditions. These findings reflect current trends in surgical decision-making. DC is generally considered a life-saving intervention for TBI patients with pronounced intracranial mass effect and refractory intracranial hypertension (14, 15). However, evidence regarding its long-term benefit remains mixed. In the CENTER-TBI study, comparative effectiveness analyses in patients with acute subdural hematoma demonstrated that institutional preference for an early surgical strategy, compared with initial conservative management, was not significantly associated with improved outcomes (OR 0.92, 95% CI: 0.77–1.09) (16). Similarly, results from the TRACK-TBI trials reported unfavorable long-term neurological outcomes in patients undergoing DC (17).

In addition, the indications for DC in geriatric patients remain undefined. In cases of epidural hematoma (EDH), emergency trephination—creating a small burr hole in the skull—can serve as a life-saving measure by providing temporary decompression of the hematoma (18, 19). Craniotomy remains the standard treatment for acute subdural hematoma (SDH), and placement of a subdural drain postoperatively facilitates removal of residual blood or cerebrospinal fluid (CSF), thereby preventing dangerous elevations in intracranial pressure (20). For selected SDH patients, particularly elderly individuals or those with unstable vital signs, preoperative trephination and drainage performed in the emergency setting may help reduce intracranial pressure, shorten operative duration, and ultimately decrease mortality (17, 21).

In current craniocerebral trauma surgery, ICP monitoring is often initiated first. Guided by ICP readings, elevated intracranial pressure can be gradually and controlledly reduced. Consequently, the incidence of acute encephalocele and contralateral delayed epidural hematoma in patients undergoing stepwise decompression is significantly lower than in those receiving conventional decompression (22). Moreover, this controlled decompression approach can also reduce the rates of vegetative state and 6-month postoperative mortality (23).

Beyond the increasing adoption of ICP monitoring, clinicians are employing more precise techniques to optimize the placement of external ventricular drain (EVD) tubes (24, 25). Minimally invasive strategies are also gaining traction; for instance, initiating a needle craniotomy for extradural hematoma using an EZ-IO device is feasible, allowing more rapid relief of intracranial pressure and providing additional time for definitive neurosurgical intervention (26). According to the Monro-Kellie doctrine, fluid removal decreases intracranial pressure, thereby improving cerebral perfusion and facilitating the delivery of osmotic agents, further supporting medical resuscitation efforts (27).

In the analysis of surgical characteristics, a higher proportion of elderly patients underwent cranial trephination and drainage, with or without ICP catheter implantation. Although a detailed subgroup analysis has not yet been performed, there is reason to believe that minimally invasive surgery may be more appropriate for elderly patients under close ICP monitoring. First, due to age-related brain atrophy, elderly patients generally have greater tolerance to intracranial hypertension and cerebral edema, and the need for decompressive craniectomy (DC) is therefore less urgent than in younger individuals. Second, elderly patients often present with multiple comorbidities, and the surgical trauma and physiological stress associated with conventional craniotomy may exacerbate their pre-existing conditions. Shen et al. (28) reported that patients aged ≥75 years had poor prognoses following traditional surgical interventions.

Wong et al. (29) prospectively collected data on consecutive trauma patients between 2001 and 2008 and demonstrated that advancing age was an unfavorable prognostic factor among patients with multiple trauma requiring neurosurgical intervention. In contrast, Lau et al. (30) prospectively enrolled consecutive patients with head trauma from 2006 to 2009 and found no statistically significant differences between age groups in 30-day mortality or recovery to baseline functional status. In the present study, which included patients admitted between 2017 and 2022, the relatively low in-hospital mortality observed among elderly patients with TBI may be explained by the increasing adoption of ICP monitoring and advanced life-support techniques, including mechanical ventilation. These advances have enabled elderly patients to better withstand the acute critical phase following injury (31).

The results of this study also indicate a higher prevalence of elderly TBI patients in a vegetative state or with severe disability, which aligns with findings from previous studies. While aggressive intensive care unit (ICU) management can improve survival in geriatric TBI patients, it is associated with an increased likelihood of discharge with severe disability (32). Elderly patients often require longer hospital stays, more home health services, and additional support during the first year post-injury (8, 33). In real-world settings, the rehabilitation and care of patients with severe disabilities or vegetative states present substantial challenges. Researchers such as Sveen et al. (34) have recommended a stronger clinical focus on older TBI patients, emphasizing the importance of continuity of care.

Several limitations should be acknowledged. First, potential selection bias may exist due to the relatively small sample size and the single-center study design. Second, the long-term prognosis of elderly patients with TBI was not evaluated. In addition, this study lacks further in-depth analyses, including the assessment of the strengths and limitations of different surgical techniques, the incorporation of the time interval between injury and surgery, and the evaluation of risk factors among patients who developed a vegetative state or severe disability. These aspects warrant further investigation.

In future research, we plan to compare patients with similar injury locations and hemorrhage types who undergo different surgical procedures, incorporate the precise interval between injury and surgery, and perform long-term follow-up to obtain a more comprehensive understanding and generate more robust conclusions. For elderly patients, future outcomes should emphasize not only survival but also changes in quality of life, which may serve as an indirect indicator of the effectiveness of local rehabilitation systems.

In summary, our study found that the in-hospital mortality rate of elderly patients with TBI decreased after surgical treatment, but there were still many patients with severe disability and vegetative state. We concluded that minimally invasive surgical procedures (such as cranial trephination and drainage) and life-support interventions (including mechanical ventilation) are crucial measures for reducing in-hospital mortality among patients with TBI. However, severe disability and a vegetative state remain conditions that require intensive care and long-term rehabilitation. For this specific patient population, greater efforts are needed to minimize disability and promote postoperative neurological recovery.

## Data Availability

The original contributions presented in the study are included in the article/supplementary material, further inquiries can be directed to the corresponding authors.
